# Associations of RANKL levels and polymorphisms with rheumatoid arthritis: A meta-analysis

**DOI:** 10.1371/journal.pone.0317517

**Published:** 2025-01-13

**Authors:** Young Ho Lee, Gwan Gyu Song

**Affiliations:** Department of Rheumatology, Korea University College of Medicine, Seoul, Korea; University of Life Sciences in Lublin, POLAND

## Abstract

**Objectives:**

This study examined the correlation between circulating receptor activator for nuclear factor-*κ*B ligand (RANKL) levels and rheumatoid arthritis (RA), and investigated the association between polymorphisms in the RANKL gene and susceptibility to RA.

**Method:**

We searched the Medline, Embase, and Cochrane databases for relevant publications up to September 2024. A meta-analysis was conducted to assess serum/plasma RANKL levels in patients with RA and controls, and to explore the relationship between RANKL rs9533156 and rs2277438 polymorphisms and RA susceptibility.

**Results:**

Ten studies encompassing 1,682 RA patients and 1,288 controls were analyzed. RANKL levels were significantly higher in RA patients compared to controls (SMD = 0.665, 95% CI = 0.290–1.040, P = 0.001). Subgroup analysis affirmed these findings’ consistency across different sample sizes and publication years. RANKL levels were positively associated with rheumatoid factor (RF) and Disease Activity Score-28 (DAS28) (RF correlation coefficient = 0.157, 95% CI = 0.028–0.282, P = 0.018; DAS28 correlation coefficient = 0.151, 95% CI = 0.125–0.370, P < 0.001). Additionally, the meta-analysis revealed significant associations between the susceptibility to RA and the RANKL rs9533156 C allele (OR = 0.609, 95% CI = 0.520–0.714, P < 0.010) as well as the rs2277438 G allele (OR = 1.206, 95% CI = 1.003–1.451, P = 0.047). These associations were consistent across homozygote comparisons and different genetic models.

**Conclusions:**

This meta-analysis underscores the elevated circulating RANKL levels in RA patients and their significant correlation with RF and DAS28. Additionally, the RANKL rs9533156 and rs2277438 polymorphisms were significantly associated with RA susceptibility.

## Introduction

Rheumatoid arthritis (RA) is a chronic autoimmune disorder that primarily affects the joints, marked by synovial inflammation, cartilage degradation, and bone erosion [1,2]. The etiology of RA involves a complex interplay between genetic predisposition and environmental factors, with numerous studies highlighting the role of pro-inflammatory cytokines and immune mediators in its pathogenesis [3,4]. Among these, the receptor activator of nuclear factor-*κ*B ligand (RANKL) is recognized as a pivotal element in bone metabolism, immune regulation, and inflammation.

RANKL, part of the tumor necrosis factor (TNF) superfamily, binds to its receptor, RANK, on osteoclast precursors to promote osteoclastogenesis and subsequent bone resorption [5]. This is particularly significant in RA, where joint destruction is a key characteristic. Besides its role in bone metabolism, RANKL is expressed by various immune cells and plays a crucial role in modulating T-cell responses, positioning it as an integral molecule at the juncture of immune regulation and bone pathology [6].

Circulating levels of RANKL have been studied in various inflammatory diseases, including RA, with inconsistent results [7–14]. Some studies have found elevated RANKL levels in RA patients compared to healthy controls, whereas others have not detected any significant differences. Additionally, genetic variations in the RANKL gene (TNFSF11) have been linked to RA susceptibility. Two particular single nucleotide polymorphisms (SNPs) in the RANKL gene, rs9533156 and rs2277438, have drawn attention because of their potential association with RA risk [12,15,16].

Given the variability among individual studies and the potential implications of genetic differences, a comprehensive meta-analysis is necessary to delineate the association between circulating RANKL levels, RANKL gene polymorphisms, and RA susceptibility. The rationale for conducting this meta-analysis stems from the established role of RANKL in bone remodeling and immune system regulation—two crucial processes implicated in the pathogenesis of RA. While individual studies have reported varying associations between circulating RANKL levels, specific RANKL polymorphisms, and RA, results have been inconsistent. This inconsistency can arise from differences in sample sizes, study populations, and methodologies. Therefore, a meta-analysis is valuable to synthesize existing data, offering a more robust estimate of these associations and potentially revealing genetic or molecular markers useful for understanding RA pathogenesis or identifying individuals at risk. This study aims to integrate existing evidence on these connections [17,18], hypothesizing that RA patients display elevated RANKL levels and that certain RANKL polymorphisms (rs9533156 and rs2277438) are linked with increased RA risk.

## Materials and methods

### Selecting relevant research and collecting data

We conducted an exhaustive literature review to identify studies measuring circulating RANKL levels (serum or plasma), investigating their association with rheumatoid arthritis (RA), and examining RANKL gene polymorphisms in RA. Searches were executed in PUBMED, EMBASE, and the Cochrane databases to find publications up to September 2024. Search terms included "RANKL," "serum OR plasma OR circulating OR blood," "polymorphism," and "rheumatoid arthritis" (Supplementary data). References cited in these studies were analyzed to discover further pertinent publications not listed in the databases. Inclusion criteria encompassed: (1) case-control, cohort, or cross-sectional studies; (2) data on RANKL levels in affected individuals and controls; and (3) analysis of RANKL gene polymorphisms in RA and control groups. Studies were excluded if they were reviews, case reports, had overlapping data, or lacked sufficient information. Extracted data included author, publication year, country, ethnicity, age, sex adjustments, participant counts, mean and standard deviation (SD) of RANKL levels, and allele and genotype frequencies of RANKL gene polymorphisms. For studies reporting data as medians, interquartile ranges, or ranges, means and SDs were estimated using recognized methods [19,20]. Two independent assessors evaluated the procedures and results, resolving any discrepancies by consensus. The quality of each study was assessed using the Newcastle-Ottawa Scale [21], and the meta-analysis conformed to the Preferred Reporting Items for Systematic Reviews and Meta-Analyses guideline [22].

### Statistical correlation analysis

A meta-analysis was conducted to explore the relationship between RANKL levels and RA, along with the impact of various RANKL gene variants. Continuous data results are presented as standard deviations from the mean with 95% confidence intervals (CIs), and dichotomous data as odds ratios (ORs) with 95% CIs. To evaluate within-study and between-study differences and heterogeneity, Cochran’s Q-statistics were utilized [23]. A heterogeneity test assessed if the same effect was consistently observed across all studies. If significant heterogeneity was found (P < 0.10), a random-effects model was employed; otherwise, a fixed-effects model was utilized. Heterogeneity was quantified using the *I*^*2*^ statistic, where *I*^*2*^ values of 25%, 50%, and 75% indicated low, moderate, and high heterogeneity, respectively [24]. Statistical analyses were conducted using the Comprehensive Meta-Analysis software (Biostat, Englewood, NJ, USA).

### Risk assessment and publication bias

Each study’s quality was assessed using the Newcastle-Ottawa Scale, achieving a maximum score of nine [21]. Scores ranging from six to nine denoted high methodological quality. A meta-regression analysis was performed using variables such as ethnicity, publication year, sample size, matching variables, data type, and study quality to explore potential sources of heterogeneity. Sensitivity analysis determined the effect of excluding individual studies on the overall effect size. Funnel plots and Egger’s linear regression test were applied to identify publication bias, with Egger’s test evaluating asymmetry in the funnel plots using standardized mean differences (SMDs) or ORs on a natural logarithmic scale.

## Results

### Studies included in the meta-analysis

**Fig 1:**
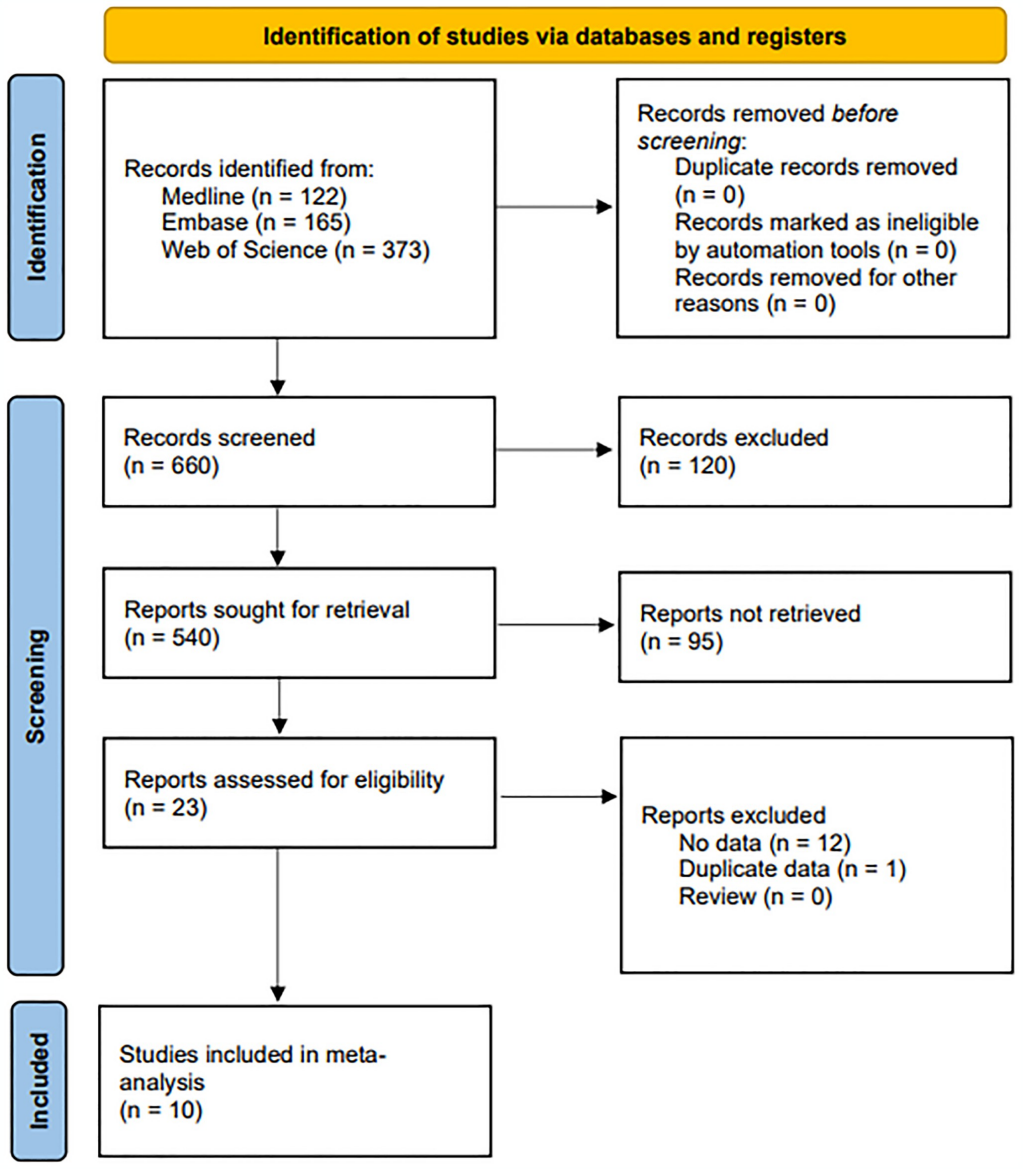
A flowchart depicting the selection process for meta-analysis studies.

We identified 660 publications using both manual and computerized search techniques. From these, 23 were selected for full-text examination based on the title and abstract, while thirteen were excluded due to lack of data or duplicate data. Consequently, ten publications met our inclusion criteria [7–16] (Fig 1). The included studies encompassed 1,682 patients with RA and 1,288 controls. The meta-analysis focused on two polymorphisms (rs9533156, and rs2277438). RANKL rs9533156 was the subject of two investigations, and rs2277438 was the subject of two. In each study, the RANKL polymorphisms in the control group were found consistent with HWE. Each study was assigned a quality grade between six and eight. Participants’ demographics and quality evaluations are presented in Table 1.

**Table 1:** Characteristics of individual studies included in the meta-analysis.

A. RANKL level										
Author	Country	Ethnicity	Cohort size (N)		RANKL level (pg/mL or mmol/L)		Statistical findings			NOS score
			Cases	Controls	Cases	Controls	SMD	Magnitude^a^	P-value	
Hussein, 2022[7]	Syria	Arab	58	30	247.92	166.57	0.412	Medium	0.069	6
Li, 2020[8]	China	Asian	60	20	336.46	103.44	0.971	Large	0.000	6
Yuce, 2017[9]	Turkey	European	17	18	2.34	1.41	0.682	Medium	0.050	6
Boman, 2017[10]	Sweden	European	407	71	0.66	0.24	0.674	Medium	0.000	7
Mohamed, 2016[11]	Egypt	Arab	172	176	10.83	2.18	1.377	Large	0.000	7
Xu, 2014[12]	China	Asian	113	100	76.69	70.13	0.172	Small	0.210	8
Ellabban, 2012[13]	Egypt	Arab	24	13	40.23	36.84	0.070	Small	0.840	6
Tan, 2010[14]	USA	European	23	24	0.64	0.26	0.853	Large	0.005	6
B. *RANKL* polymorphisms										
Author	Country	Ethnicity	Cohort size (N)		*RANKL* polymorphisms tested		Statistical findings (p-value)			NOS score
			Cases	Controls						
Arturo, 2024[15]	Mexico	Latin American	94	134	Rs9533156		Rs9533156 (p = 0.016)			6
Xu, 2014[12]	China	Asian	200	188	Rs2277438		RS2277438 (NS)			7
Assmann, 2010[16]	Germany	European	514	514	Rs9533156, rs2277438		Rs9533156 (p = 0.047), rs2277438 (NS)			7

*SMD*, standard mean difference;

^a^ Magnitude of Cohen’s d effect size where 0.2 to 0.5 is a small effect, 0.5 to 0.8 is a medium effect, and ≥ 0.8 is a large effect,

N: Number, NOS: Newcastle-Ottawa Scale.

*NS*, not significant, NOS: Newcastle-Ottawa Scale.

### Meta-analysis of the correlation between circulating RANKL concentrations and RA

**Fig 2:**
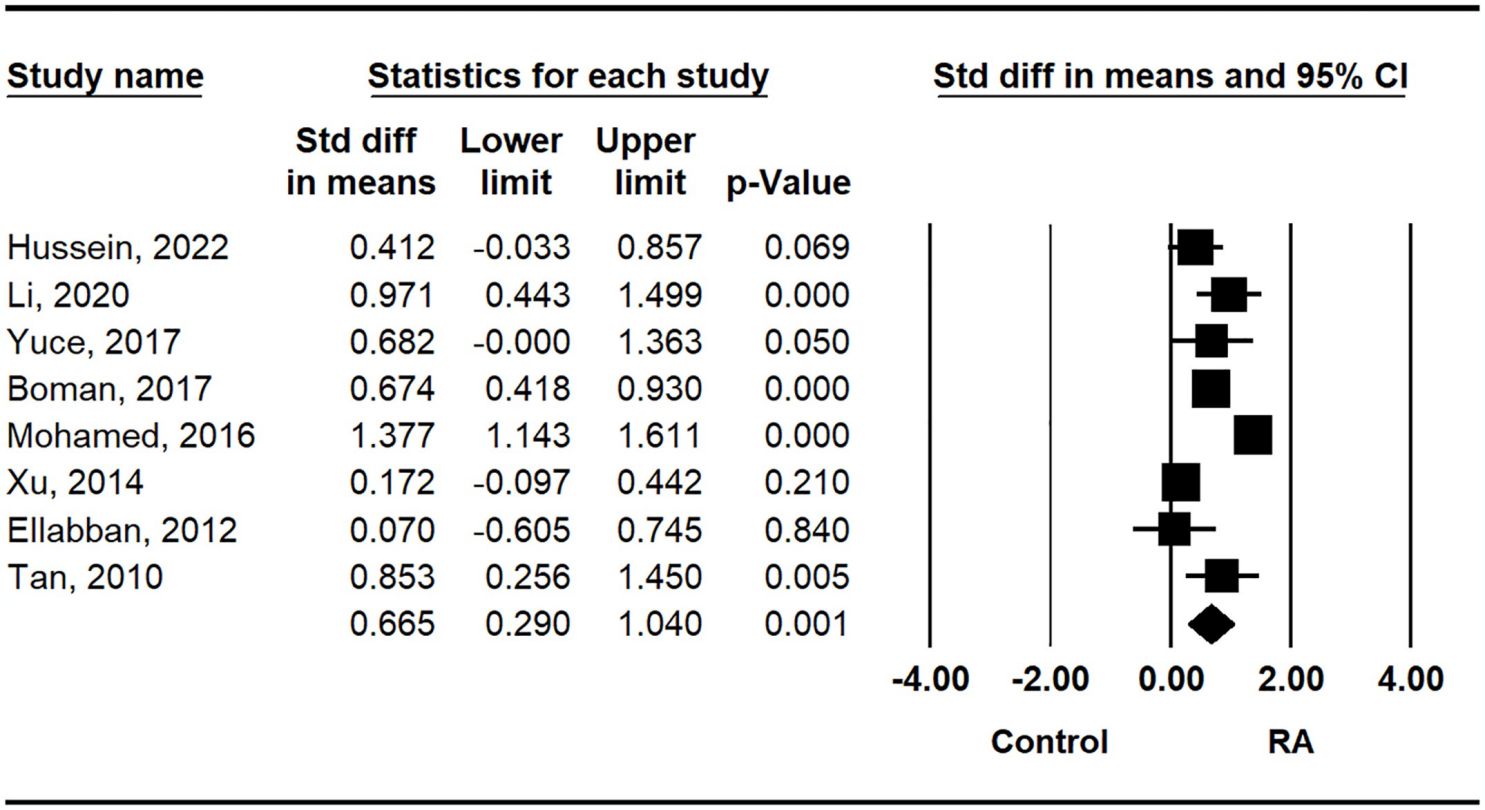
Meta-analysis of the correlation between circulating RANKL concentrations and RA.

We found that RANKL levels in the RA group were significantly higher than those in the control group (SMD = 0.665, 95% CI = 0.290–1.040, P = 0.001) (Table 2 and Fig 2). Meta-analysis was conducted on RA patients grouped by ethnicity, age, sex adjustments, sample size, and publication year in each subgroup. Europeans exhibited higher RANKL levels compared to Arab and Asian populations according to ethnicity-based stratification (SMD = 0.700, 95% CI = 0.477–0.922, P < 0.001) (Table 2). Subgroup analysis by adjustment also revealed significantly higher RANKL levels in the RA group (Table 2), irrespective of the adjustment variables. Significantly higher RANKL levels in the RA group were observed in both the small (N = 100) and large (N ≥ 100) sample size subgroups (Table 2). Furthermore, subgroup analysis by publication year indicated significantly elevated RANKL levels in both recent and older studies (Table 2).

**Table 2:** Meta-analysis of the association between circulating RANKL levels and RA.

Groups	Population	No. of studies	Test of association			Test of heterogeneity		
			SMD*	95% CI	*p* val.	Model	*p*-val.	*I* ^*2*^
All	Overall	8	0.665	0.290–1.010	0.001	R	< 0.001	86.6
Ethnicity	European	3	0.700	0.477–0.922	< 0.001	F	0.863	0
	Arab	3	0.659	-0.184–1.5021	0.126	R	< 0.001	91.4
	Asian	2	0.538	-0.242–1.318	0.176	R	0.008	85.6
Matched for age and sex	Yes	6	0.633	0.179–1.088	0.006	R	< 0.001	90.4
	No	2	0.779	0.329–1.228	0.001	F	0.711	0
Sample size	N ≥ 100	3	0.744	0.051–1.437	0.035	R	< 0.001	95.5
	N < 100	5	0.607	0.355–0.858	< 0.001	F	0.223	29.7
Publication year	Recent (≥ 2015)	5	0.844	0.439–1.250	< 0.001	R	< 0.001	82.9
	Old (< 2015)	3	0.262	0.031–0.483	0.026	R	0.106	55.5

IL-6: Interleukin-6, SMD: Standardized mean difference;

*magnitude of Cohen’s d effect size (SMD): 0.2–0.5, small effect; 0.5–0.8, medium effect; ≥0.8, large effect;

N: Number, R: Random effects model; NA: Not available.

#### Meta-analysis of the correlation between circulating RANKL levels and RF and activity in RA

**Fig 3:**
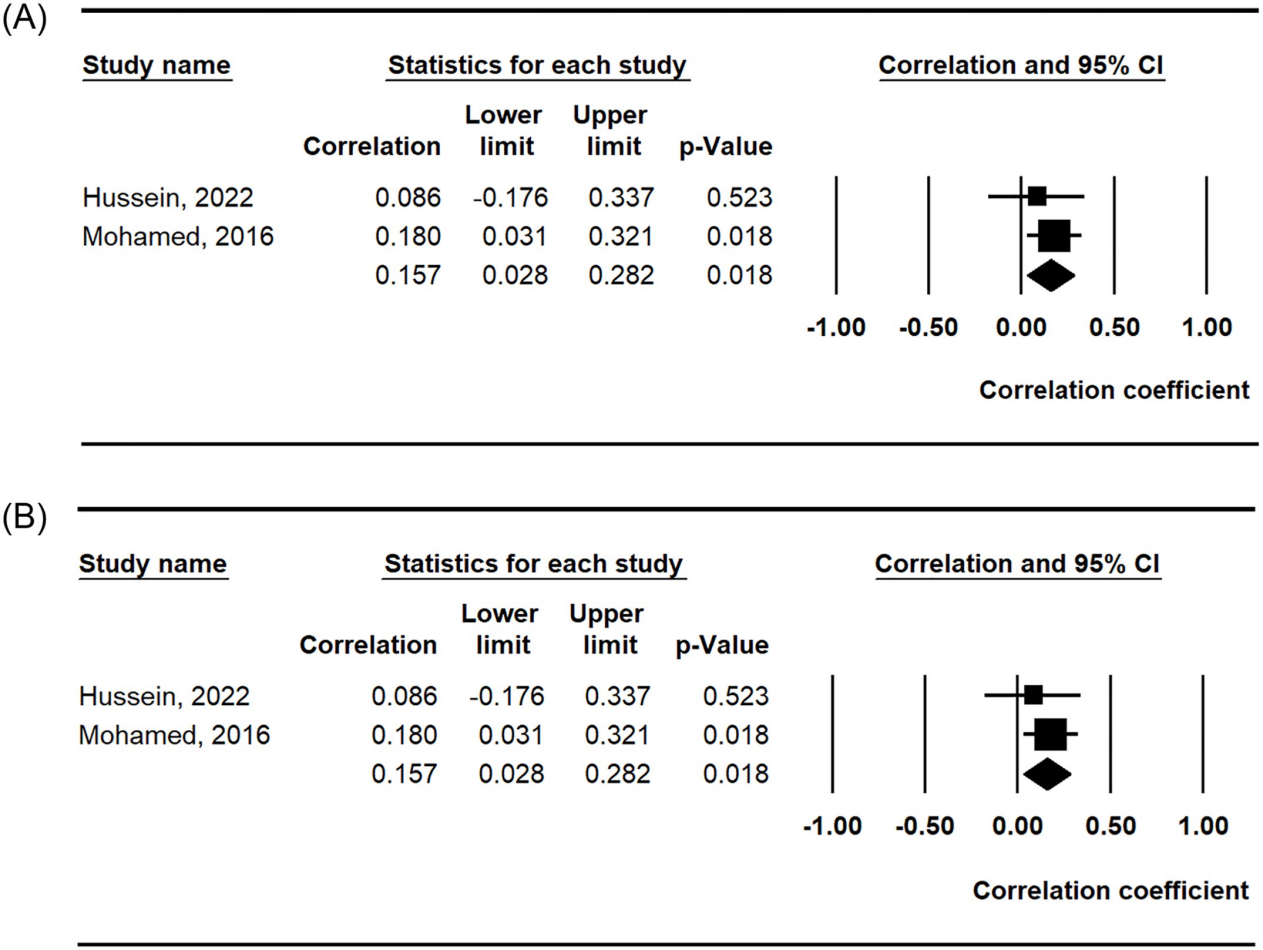
Meta-analysis of the correlation coefficient between RANKL levels and RF (A), and DAS28 (B).

Meta-analysis of correlation coefficients revealed no association between calprotectin levels and ESR (Table 3 and Fig 3). However, this meta-analysis showed that calprotectin levels were positively correlated with RF and DAS28 (correlation coefficient of RF = 0.157, 95% CI = 0.028–0.282, P = 0.018; correlation coefficient of DAS28 = 0.151, 95% CI = 0.125–0.370, P < 0.001) (Table 3 and Fig 3).

**Table 3:** Meta-analysis of the correlation coefficient between RANKL level and ESR, CRP, RF, and DAS28 in RA.

Parameters	No. of studies	Test of association			Test of heterogeneity		
		Correlation coefficient	95% CI	*p*-value	Model	*p*-value	I^*2*^
ESR	2	0.100	-0.031–0.227	0.133	F	0.596	0
CRP	2	0.112	-0.134–0.344	0.373	R	0.099	63.2
RF	2	0.157	0.028–0.282	0.018	F	0.537	0
DAS28	2	0.151	0.125–0.370	< 0.001	F	0.155	50.6

RA: Rheumatoid arthritis, RANKL: Receptor activator of nuclear factor-κB ligand, ESR: Erythrocyte sedimentation rate, CRP: C-reactive protein, RF: Rheumatoid factor, DAS28: DAS based on 28 joints, CI: Confidence interval, F: Fixed effects model, R: Random effects model.

### RANKL rs9533156 and rs2277438 polymorphisms and RA susceptibility

**Fig 4:**
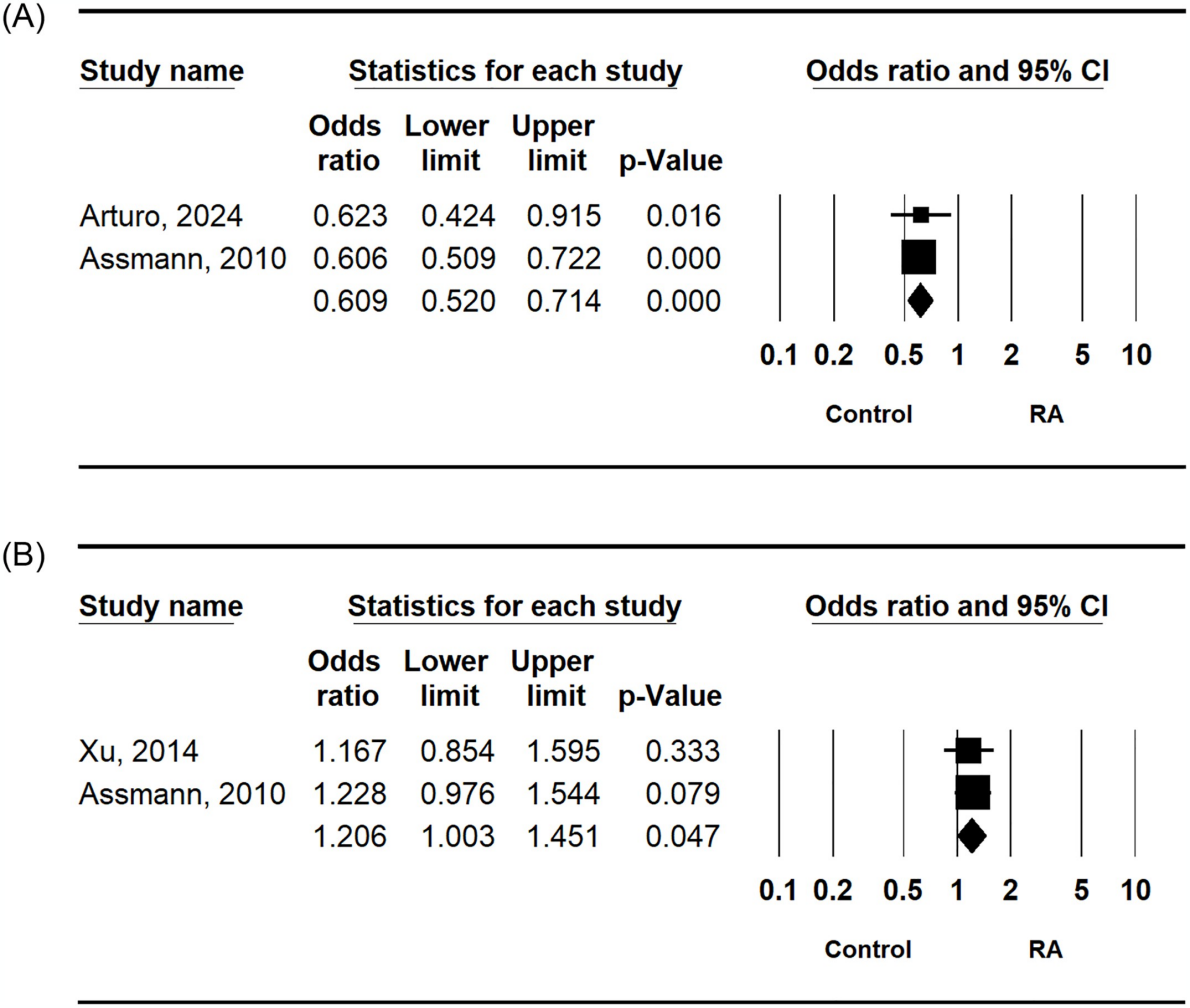
Odds ratios and 95% confidence intervals for studies and aggregated data showing the allelic association between the RANKL polymorphisms at rs9533156 (A), and rs2277438 (B) and RA.

This meta-analysis demonstrated the significant association between RA and the RANKL rs9533156 C allele (OR = 0.609, 95% CI = 0.520–0.714, P < 0.010) (Table 3 and Fig 4). Both homozygote contrast and dominant models of RANKL rs9533156 polymorphism in RA showed correlations (Table 3). This meta-analysis also highlighted the significance of the association between RA and the RANKL rs2277438 G allele (OR = 1.206, 95% CI = 1.003–1.451, P = 0.047) (Table 3 and Fig 4). Homozygote contrast and recessive models of RANKL rs2277438 polymorphism in RA exhibited correlations (Table 3).

### Heterogeneity and publication bias

Heterogeneity in the meta-analysis of RANKL levels in RA patients was observed (Table 2). Significant heterogeneity was noted considering factors such as sample size, publication year, data format, and matching characteristics (p < 0.05); however, meta-regression tests indicated that study quality and ethnicity did not significantly impact heterogeneity. The meta-analysis’s reliability was confirmed by sensitivity analysis, which showed no significant effects from including or excluding studies. Furthermore, meta-analyses investigating RANKL polymorphisms discovered no between-study heterogeneity (Table 4). Publication bias emerges as a concern in meta-analyses with an excess of positive studies, but no publication bias was detected in this analysis (no asymmetry in the funnel plot; p-values for Egger’s test were above 0.05).

**Table 4:** Meta-analysis of the associations between the RANKL rs9533156 and rs2277438 polymorphism and RA.

Polymorphism	Population	No. of studies	Test of association			Test of heterogeneity		
			OR	95% CI	*p*-val	Model	*p*-val	*I* ^*2*^
Rs9533156 C vs. T	Overall	2	0.609	0.520–0.714	< 0.001	F	0.899	0
CC vs. CT + TT (recessive)	Overall	2	0.783	0.594–1.032	0.082	F	0.903	0
CC + CT vs. TT (dominant)	Overall	2	0.773	0.608–0.983	0.035	F	0.473	0
CC vs. CT	Overall	2	0.697	0.507–0.958	0.026	F	0.844	0
Rs2277438 G vs. A	Overall	2	1.206	1.003–1.451	0.047	F	0.713	0
GG vs. GA + AA (recessive)	Overall	2	1.787	1.065–3.000	0.028	F	0.177	45.0
GG + GA vs. AA (dominant)	Overall	2	1.164	0.933–1.452	0.179	F	0.746	0
GG vs. AA	Overall	2	1.879	1.110–3.180	0.019	F	0.243	26.5

OR: Odds ratio, CI: Confidence interval, F: Fixed effects model.

## Discussion

This meta-analysis provides strong evidence of RANKL’s involvement in the pathogenesis of RA. Analysis of ten studies demonstrates a consistent elevation in circulating RANKL levels among RA patients compared to controls. The standardized mean difference (SMD = 0.665, P = 0.001) suggests a moderate yet significant rise in RANKL levels, supporting its potential role as a biomarker for RA disease activity and progression. The relationship between RANKL and RA pathogenesis is complex. RANKL is essential for osteoclast differentiation and activation, key processes for bone resorption in RA [6]. Additionally, increased RANKL levels in RA patients may directly contribute to osteoclastic activity responsible for bone erosions observed in RA-affected joints [25]. Moreover, the role of RANKL in T-cell activation and dendritic cell survival underscores its broader immunological functions, potentially exacerbating the chronic inflammatory environment in RA [26].

The observed positive correlations between circulating RANKL levels and key clinical parameters like RF and DAS28 further underscore RANKL’s significance in assessing RA disease severity. The correlation coefficients of RANKL with RF (0.157, P = 0.018) and DAS28 (0.151, P < 0.001) highlight its potential as a marker for both disease presence and inflammatory burden. These findings align with previous studies linking elevated RANKL levels to increased radiographic joint damage in RA patients, suggesting RANKL’s potential predictive role in monitoring RA progression.

Beyond circulating RANKL levels, this study also investigated the association between RANKL gene polymorphisms and RA susceptibility. The meta-analysis revealed significant associations between the rs9533156 C allele and reduced RA risk (OR = 0.609, P < 0.010), as well as the rs2277438 G allele and increased RA risk (OR = 1.206, P = 0.047). These results propose that genetic variations in the RANKL gene may influence individual susceptibility to RA, potentially through different regulatory effects on RANKL expression or function.

The protective role against RA linked with the rs9533156 C allele is particularly noteworthy. This SNP is located in a regulatory region of the RANKL gene, and it is theorized that the C allele may decrease RANKL expression or activity, attenuating the inflammatory and osteoclastic processes integral to RA pathogenesis. In contrast, the rs2277438 G allele, associated with increased RA risk, may amplify RANKL expression, thereby facilitating osteoclastogenesis and immune activation typical of RA. These genetic correlations align with findings from other autoimmune diseases where RANKL polymorphisms have been associated with disease susceptibility. However, the exact mechanisms through which these polymorphisms affect RA risk are yet to be defined. Functional studies should aim to verify whether these SNPs modify RANKL gene expression, protein functionality, or interactions with other immune mediators. Future research should focus on unveiling the molecular mechanisms through which RANKL gene polymorphisms influence RA susceptibility and on developing targeted therapies that intervene in the RANK/RANKL/osteoprotegerin pathway. Given RANKL’s role in bone resorption, inhibitors of the RANKL-RANK interaction, such as denosumab, may prove beneficial in preventing joint damage in RA patients.

A previous meta-analysis by Yang et al. examined the association between RANKL polymorphisms and RA risk but did not cover circulating RANKL levels comprehensively across populations [27]. Their study specifically analyzed polymorphisms across RANK, RANKL, and osteoprotegerin (OPG) genes and found some SNPs linked to RA susceptibility. However, the analysis did not include correlations between circulating RANKL levels and RA disease activity or biomarkers such as RF or DAS28. The current meta-analysis adds value by addressing both circulating RANKL levels and specific RANKL polymorphisms (rs9533156 and rs2277438) in relation to RA susceptibility. It also explores the relationship between RANKL levels and disease severity, offering a more detailed understanding of RANKL’s role in RA pathogenesis.

Elevated circulating levels of RANKL and genetic polymorphisms in the RANKL gene play a significant role in the pathogenesis of RA through various mechanisms. First, RANKL binds to its receptor, RANK, on osteoclast precursors, activating the NF-κB pathway [28]. This process induces osteoclast differentiation and bone resorption, which are central to RA-related joint damage. Second, pro-inflammatory cytokines like IL-29 upregulate RANKL expression in synovial fibroblasts via MAPK signaling, amplifying inflammation and joint erosion [29]. Third, SNPs in RANKL affect RANKL activity and susceptibility to RA, with variations potentially influencing RANKL production and exacerbating bone erosion and inflammation [30]. Lastly, RANKL activity is regulated by osteoprotegerin (OPG), a decoy receptor that inhibits the interaction between RANK and RANKL [31]. An imbalance in this regulatory mechanism, often driven by RANKL polymorphisms, further promotes osteoclast activation in RA.

To date, genome-wide association studies (GWAS) directly linking RANKL polymorphisms, such as rs9533156 and rs2277438, with rheumatoid arthritis (RA) have been limited. While GWAS have identified numerous loci associated with RA susceptibility, especially within immune-related pathways, most studies have focused on genes within the major histocompatibility complex (MHC) region, such as *HLA-DRB1*, or on non-HLA loci like *PTPN22* and *STAT4*. Although this meta-analysis offers important insights into the role of RANKL in RA, it also presents several limitations. First, the diversity among the included studies, particularly in terms of study populations, sample sizes, and RANKL measurement methods, may introduce variability into the results. Moreover, the potential influence of confounding factors such as medication use, disease duration, and comorbidities on RANKL levels cannot be completely dismissed. Second, denosumab, as a direct RANKL inhibitor, has primarily shown effects on suppressing joint destruction rather than RA disease activity. Our study did not aim to propose circulating RANKL levels as a direct surrogate for disease activity in RA. Rather, we investigated RANKL as a potential biomarker that reflects specific pathological processes. Third, findings on genetic polymorphisms are preliminary, and their translational relevance to clinical practice remains unclear. In previous GWAS analyses of RA, data relating to these RANKL gene polymorphisms and RA disease susceptibility have not been clearly shown [32]. Potential biases, such as population-specific allele frequencies and heterogeneity in study designs, could influence meta-analyses and genetic association studies. However, this meta-analysis had numerous strengths. To our knowledge, it is the first to concurrently explore two related aspects: circulating RANKL levels and polymorphisms in RANKL-encoding genes among RA patients. While individual study sample sizes ranged from 17 to 514 RA participants, our combined analysis encompassed a total of 1,682 patients with RA. Furthermore, by aggregating results from multiple independent studies, we enhanced the statistical power and accuracy of the analysis, yielding findings more robust than those of individual studies alone [33–35].

In conclusion, this meta-analysis has demonstrated that patients with RA exhibit significantly elevated circulating RANKL levels compared to healthy controls, and these levels are positively correlated with RF and DAS28. Additionally, genetic polymorphisms in the RANKL gene, specifically rs9533156 and rs2277438, are associated with increased susceptibility to RA. These insights highlight the potential of RANKL as a therapeutic target.

## Supporting information

S1 Checklist PRISMA 2020 checklist. (DOCX)

S2 Checklist Meta-analysis on genetic association study checklist. (DOCX)

S1 Table Search strategy. (DOCX)

S2 Table All data extracted in primary studies. (XLSX)
