# Supplementary material for: Associations of RANKL levels and polymorphisms with rheumatoid arthritis: A meta-analysis
Source: PLoS One. 2025 Jan 13;20(1):e0317517. doi: 10.1371/journal.pone.0317517 (PMC11729962; doi:10.1371/journal.pone.0317517)
Supplement: S1 Table — (DOCX) [file pone.0317517.s003.docx]

**Search strategy**

**MEDLINE (n = 122)**

(('rheumatoid arthritis'/exp OR 'beauvais disease' OR 'arthritis deformans' OR 'arthritis, rheumatoid' OR 'arthrosis deformans' OR 'chronic articular rheumatism' OR 'chronic polyarthritis' OR 'chronic rheumatoid arthritis' OR 'disease, beauvais' OR 'infantile rheumatoid arthritis' OR 'inflammatory arthritis' OR 'polyarthritis rheumatica' OR 'polyarthritis, primary chronic' OR 'primary chronic polyarthritis' OR 'rheumarthritis' OR 'rheumatic arthritis' OR 'rheumatic polyarthritis' OR 'rheumatism, chronic articular' OR 'rheumatoid arthritis' OR 'rheumatoid polyarthritis') AND ('osteoclast differentiation factor'/exp OR 'cd254 antigen' OR 'odf' OR 'opgl' OR 'rank ligand' OR 'rankl' OR 'tnfsf 11 protein' OR 'tnfsf11 protein' OR 'antigen cd254' OR 'osteoclast differentiation factor' OR 'osteoprotegerin ligand' OR 'protein tnfsf 11' OR 'protein tnfsf11' OR 'receptor activator of nf kappa b ligand' OR 'receptor activator of nuclear factor kappa b ligand' OR 'tumor necrosis factor ligand superfamily member 11' OR 'tumor necrosis factor related activation induced cytokine' OR 'tumour necrosis factor ligand superfamily member 11' OR 'tumour necrosis factor related activation induced cytokine') AND ('polymorphism'/exp OR 'genetic polymorphism'/exp OR 'genetic polymorphism' OR 'polymorphism (genetics)' OR 'polymorphism, genetic') AND 'osteoclast differentiation factor'/exp)

**Embase (n = 165)**

(('rheumatoid arthritis'/exp OR 'beauvais disease' OR 'arthritis deformans' OR 'arthritis, rheumatoid' OR 'arthrosis deformans' OR 'chronic articular rheumatism' OR 'chronic polyarthritis' OR 'chronic rheumatoid arthritis' OR 'disease, beauvais' OR 'infantile rheumatoid arthritis' OR 'inflammatory arthritis' OR 'polyarthritis rheumatica' OR 'polyarthritis, primary chronic' OR 'primary chronic polyarthritis' OR 'rheumarthritis' OR 'rheumatic arthritis' OR 'rheumatic polyarthritis' OR 'rheumatism, chronic articular' OR 'rheumatoid arthritis' OR 'rheumatoid polyarthritis') AND ('osteoclast differentiation factor'/exp OR 'cd254 antigen' OR 'odf' OR 'opgl' OR 'rank ligand' OR 'rankl' OR 'tnfsf 11 protein' OR 'tnfsf11 protein' OR 'antigen cd254' OR 'osteoclast differentiation factor' OR 'osteoprotegerin ligand' OR 'protein tnfsf 11' OR 'protein tnfsf11' OR 'receptor activator of nf kappa b ligand' OR 'receptor activator of nuclear factor kappa b ligand' OR 'tumor necrosis factor ligand superfamily member 11' OR 'tumor necrosis factor related activation induced cytokine' OR 'tumour necrosis factor ligand superfamily member 11' OR 'tumour necrosis factor related activation induced cytokine') AND ('polymorphism'/exp OR 'genetic polymorphism'/exp OR 'genetic polymorphism' OR 'polymorphism (genetics)' OR 'polymorphism, genetic') AND 'osteoclast differentiation factor'/exp)

**Web of Science (n = 373)**

Rheumatoid arthritis (all field) AND (RANK OR RANKL polorphism) (all field)
